# Global clinical landscape of stem cells in CKD: clinical trials, challenges, and opportunities

**DOI:** 10.1097/MS9.0000000000004306

**Published:** 2025-11-22

**Authors:** Meijun Liu, Lu Bian, Haipeng Sun, Peng Zhang, Cuizhen Liu, Liya Wang

**Affiliations:** a Department of Nephrology, High tech Campus, Tai’an Central, Hospital Affiliated to Qingdao University, Taian, Shandong Province, China; b Department of Spine Surgery, Taian Central Hospital Affiliated to Qingdao University, Taian City, Shandong Province, China

*Dear Editor,*

Chronic kidney disease (CKD) has emerged as a leading chronic condition that poses a significant threat to global human health. CKD is characterized by an insidious onset, with most patients requiring long-term renal replacement therapy upon progression to end-stage renal disease. Consequently, the development of effective therapeutic strategies and novel pharmacological interventions prior to the initiation of dialysis has become a central focus in global nephrology research. With the synergistic advancement of cell biology and clinical medicine, the therapeutic potential and safety of mesenchymal stem cell (MSC) therapy in the treatment of chronic diseases have been increasingly recognized. Accumulating evidence demonstrates that MSCs possess the capacity to modulate immune responses, facilitate tissue repair, and promote cellular regeneration^[1^],[^2]^.

Numerous global studies have explored MSC therapy for CKD. To guide future treatment strategies, we evaluated the current status of clinical trials assessing the safety and efficacy of MSCs in CKD. We systematically searched the Trialtrove database (https://pharma.id.informa.com) using keywords including “chronic kidney disease”or“CKD”or“chronic renal failure”or“CKF,” and terms related to stem cell therapy such as “stem cells”or“cell therapy”or“mesenchymal stem cells”or“MSC”or“induced pluripotent stem cells”or“iPSC.” The search was updated to 20 June 2025. After rigorous analysis and screening, 51 trials meeting inclusion criteria were selected for detailed evaluation. Two independent researchers reviewed and verified the data to ensure accuracy. The study followed the 2025 TITAN guidelines, and no AI tools were used in any stage of the research or writing^[3]^.

Our analysis has identified several key findings. Firstly, the initial trial was launched in 2008, followed by notable increases in trial initiation in 2014, 2019, and 2023. As of June 2025, the number of clinical trials continues to rise (Fig. 1A), reflecting a growing global interest in MSC therapies for CKD and underscoring the promising trajectory of this treatment modality. As illustrated in Figure 1A, the majority of trials are in Phase I (41.2%) and combined Phase I/II (25.5%), with six trials (11.8%) having advanced to Phase IV. These findings support the feasibility and safety of MSC therapy in CKD and indicate that the field remains in its early developmental stages. As shown in Figure 1B, among the 51 trials analyzed, most were conducted in China (27.4%) and the United States (25.5%), followed by South Korea (7.8%), and Iran (9.8%). The concentration of studies in economically developed regions highlights significant geographical disparities. Future research should aim to expand clinical investigations into low- and middle-income countries to address these imbalances. Among the top five countries with the highest number of trials, most remain in Phase I. Notably, China, the United States, and South Korea exhibit a higher proportion of Phase IV trials, likely due to their stronger economic resources and advanced technological infrastructure (Fig. 1C). Currently, 23 trials (45.1%) have been completed (Fig. 1D), with six (11.8%) reporting positive outcomes (Table 1). Additionally, 11 trials (21.6%) are ongoing, while six (11.8%) have been terminated, primarily due to insufficient funding and challenges in patient recruitment. Seven trials (13.7%) are currently in the planning phase (Fig. 1C). With regard to sponsorship, academic institutions sponsored 54.9% of the trials, whereas industry-sponsored trials accounted for 17.6% (Fig. 1E).

**Figure 1.**
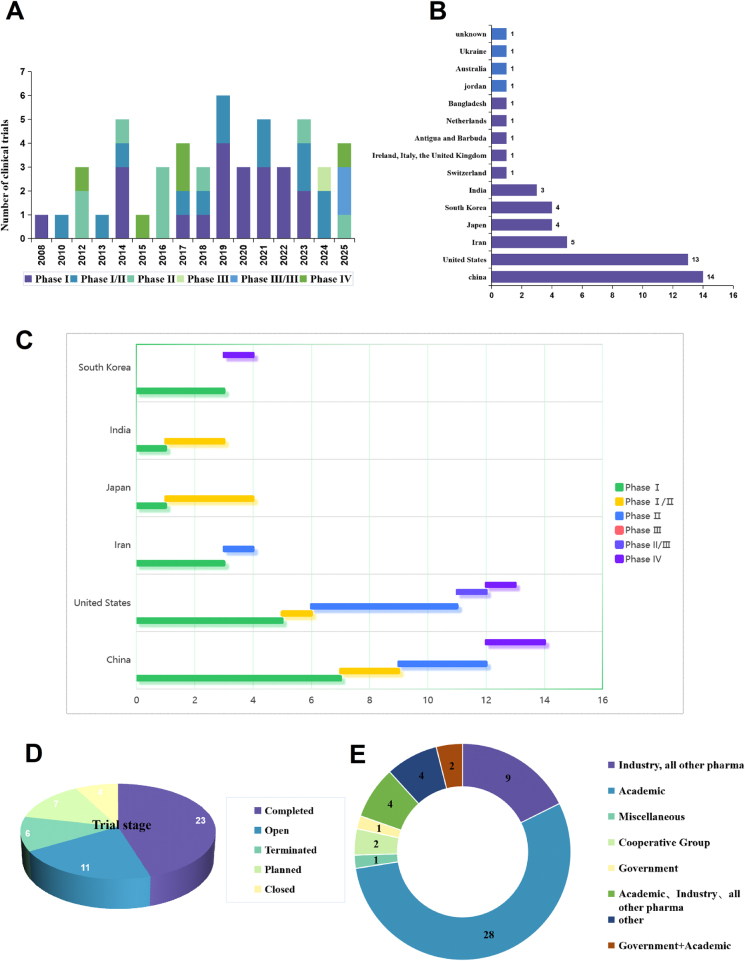
Overview of clinical trials on stem cell therapy for chronic kidney disease. (A) Temporal trends of clinical trials by start years and trial phase distribution. (B)Country distribution of clinical trials. (C) The distribution of clinical trial phases in different countries (Top 5 in the number of experiments). (D) Distribution of trial phases and trial statuses of stem cell therapy for CKD. (E) Sponsor type distribution of clinical trials.

**Table 1:** Specific details of positive and negative result items in completed clinical trials

Trial ID	Title	Stage	Countries	Patient segment	No. of patients
**Completed, positive outcome**					
414647	G-CSF-mobilized peripheral blood-derived autologous CD34+ cell transplantation for patients with chronic kidney disease	I/II1	Japan	CKD, Stage 3; CKD, Stage 4	4
371725	Open-label, Multiple-center, Dose-Escalation Study to Evaluate the Safety and Tolerability of ADR-001 for the Treatment for Immunoglobulin A (IgA) Nephropathy	I	Japan	CKD, Stage 1; CKD, Stage 2; CKD, Stage 3; Unspecified IgA Nephropathy	9
351863	Application of peripheral blood-derived stem cell/progenitor cell (CD34+) therapy on chronic kidney disease: Phase 2 clinical trial	II	Taiwan, China	CKD, Stage 3; CKD, Stage 4; End-Stage Renal Disease (ESRD); Hypertensive Nephropathy	52
211167	Evaluation the Effect of Mesenchymal Stem Cells Transplantation in Patients With Chronic Renal Failure Due to Autosomal Dominant Polycystic Kidney Disease	I	Iran	Autosomal dominant (ADPKD), unspecified; CKD, Stage 3; CKD, Stage 4	6
179928	Combined Haploidentical Reduced Intensity Bone Marrow and Kidney Transplantation for Patients with Chronic Kidney Disease and Advanced Hematological Disorders	II	United States	Adults; kidney transplant; solid organ transplant; transplant – stem cell	6
185497	A Randomized, Controlled, Dose-Escalation Pilot Study to Assess the Safety and Efficacy of a Single Intravenous Infusion of Allogeneic Mesenchymal Precursor Cells (MPCs) in Subjects With Diabetic Nephropathy and Type 2 Diabetes	I/II1	Australia	CKD, Stage 3; CKD, Stage 4; diabetic nephropathy	30
**Completed, Negative outcome**					
259411	Evaluation of effects of injected autologous mesenchymal stem cells on kidney function in patients with chronic kidney disease	II	Iran	CKD, Stage 4	14

As presented in Table 2, the majority of clinical trials (56.9%) have targeted CKD stage 3 and stage 4. This distribution may be attributed to the fact that stages 1 and 2 typically represent early phases of kidney disease, where conventional pharmacological interventions are often effective. In contrast, stage 5 is characterized by a high-toxicity uremic environment, which may impair the differentiation capacity of stem cells and hinder angiogenesis^[4]^. MSCs have been proven to release growth factors and cytokines that regulate inflammation and promote tissue repair through paracrine signaling. Therefore, for patients in stages 3 and 4 of CKD, MSCs can stabilize the slope of the decline in glomerular filtration rate and delay the initiation of dialysis. Among the completed trials with positive results, four positive results came from patients in stage 3 and 4 of CKD (Table 1). Table 2 further reveals that trials focusing on diabetic nephropathy or combined diabetic nephropathy dominate (37.3%), with the remaining trials mainly covering hypertensive nephropathy (17.6%), kidney transplantation (9.8%), rare diseases (6.0%), IgA (2.0%), etc.

**Table 2:** Research distribution in different stages of chronic kidney disease and primary diseases

Characteristic	*N* = 51
Patient Segment	
CKD 1-3	3
CKD 1-4	1
CKD 2-3	2
CKD 2-4	1
CKD 3	7
CKD 3-4	21
CKD 3-4、End-Stage Renal Disease	3
CKD 4	1
CKD 4、End-Stage Renal Disease (ESRD)	2
End-stage renal disease (ESRD)	1
Alport syndrome	2
Kidney transplant	5
CKD、Unspecified	2
Disease	
Diabetic kidney disease	14
Hypertensive nephropathy	4
The primary disease was not mentioned	18
Alport	2
After kidney transplantation	5
Hypertension complicated with diabetes	5
IgA	1
Adpkd	1
Unknown	1

MSCs accounted for the majority of stem cell interventions in the included studies (76.5%), with primary sources including bone marrow (BM)-MSCs, adipose tissue (AT)-MSCs, and umbilical cord blood (UC)-MSCs. MSCs derived from different tissues exhibit distinct biological characteristics. For example, UC-MSCs demonstrate superior proliferation capacity, extended lifespan, and enhanced differentiation potential^[5]^. In contrast, BM-MSCs and AT-MSCs offer the advantage of more straightforward isolation and collection procedures. Notably, Amimestrocel, a mesenchymal stem cell therapy product developed by JCR Pharmaceuticals (Japan), is currently undergoing phase 2 clinical evaluation in China, which may serve as a critical step toward the broader clinical application of MSCs in CKD. Beyond conventional MSCs, we identified four clinical trials (7.8%) utilizing G-CSF-mobilized autologous peripheral blood-derived CD34^+^ cells. Other stem cell modalities included hematopoietic stem cell therapies (5.9%), induced pluripotent stem cell therapies (3.9%), and gene-modified autologous hematopoietic stem cell transplantation (1.95%). Only two trials (3.9%) involved allogeneic stem cell therapy. Additionally, we identified two studies (3.9%) from South Korea investigating urine-derived stem cells. Urine-derived stem cells show promising potential for promoting regeneration of urinary system tissues.

Our study offers an overview of current MSC-based therapies for CKD. Clinical evidence remains limited by small sample sizes, short follow-up periods, and uncertainty about long-term effects. However, many completed trials have shown positive results, indicating a promising direction for CKD treatment. Since CKD involves diverse causes and has a clear staging system, future research should focus on larger trials, evaluating treatment differences across disease types, long-term safety, and standardized protocols. Early-stage CKD also requires more attention. We hope these findings will support the development and clinical application of new therapies for chronic renal failure.

## Data Availability

The datasets generated and analyzed during the current study are available in the INFORMA database (https://pharma.id.informa.com/).

## Research registration unique identifying number (UIN)

ChiCTR2400083681.
